# α_1A_-Adrenergic Receptor-Directed Autoimmunity Induces Left Ventricular Damage and Diastolic Dysfunction in Rats

**DOI:** 10.1371/journal.pone.0009409

**Published:** 2010-02-24

**Authors:** Katrin Wenzel, Gerd Wallukat, Fatimunnisa Qadri, Norbert Hubner, Herbert Schulz, Oliver Hummel, Florian Herse, Arnd Heuser, Robert Fischer, Harald Heidecke, Friedrich C. Luft, Dominik N. Muller, Rainer Dietz, Ralf Dechend

**Affiliations:** 1 Medical Faculty of the Charité, Berlin, Germany; 2 Experimental and Clinical Research Center and Max-Delbrück Center for Molecular Medicine, Berlin, Germany; 3 CellTrend, Luckenwalde, Germany; 4 HELIOS Clinic-Berlin, Franz-Volhard Clinic, Berlin, Germany; University of Illinois at Chicago, United States of America

## Abstract

**Background:**

Agonistic autoantibodies to the α_1_-adrenergic receptor occur in nearly half of patients with refractory hypertension; however, their relevance is uncertain.

**Methods/Principal Findings:**

We immunized Lewis rats with the second extracellular-loop peptides of the human α_1A_-adrenergic receptor and maintained them for one year. α_1A_-adrenergic antibodies (α_1A_-AR-AB) were monitored with a neonatal cardiomyocyte contraction assay by ELISA, and by ERK1/2 phosphorylation in human α_1A_-adrenergic receptor transfected Chinese hamster ovary cells. The rats were followed with radiotelemetric blood pressure measurements and echocardiography. At 12 months, the left ventricles of immunized rats had greater wall thickness than control rats. The fractional shortening and dp/dt_max_ demonstrated preserved systolic function. A decreased E/A ratio in immunized rats indicated a diastolic dysfunction. Invasive hemodynamics revealed increased left ventricular end-diastolic pressures and decreased dp/dt_min_. Mean diameter of cardiomyocytes showed hypertrophy in immunized rats. Long-term blood pressure values and heart rates were not different. Genes encoding sarcomeric proteins, collagens, extracellular matrix proteins, calcium regulating proteins, and proteins of energy metabolism in immunized rat hearts were upregulated, compared to controls. Furthermore, fibrosis was present in immunized hearts, but not in control hearts. A subset of immunized and control rats was infused with angiotensin (Ang) II. The stressor raised blood pressure to a greater degree and led to more cardiac fibrosis in immunized, than in control rats.

**Conclusions/Significance:**

We show that α_1A_-AR-AB cause diastolic dysfunction independent of hypertension, and can increase the sensitivity to Ang II. We suggest that α_1A_-AR-AB could contribute to cardiovascular endorgan damage.

## Introduction

α_1_-adrenergic receptors (α_1_-AR) mediate vascular smooth muscle cell (VSMC) contraction, cardiac inotropy, hypertrophy, and remodeling [1]. Others and we have described agonistic autoantibodies against the α_1_-AR in hypertensive patients [2], [3], [4], [5]. We found earlier that α_1_-AR-autoantibody immunoadsorption reduced blood pressure in patients with refractory hypertension [5]. In that study, rabbit or patient-derived α_1A_-AR-autoantibodies were purified with chromatography and characterized by epitope mapping and surface plasmon resonance measurements. Phospholipase A2 group IIA (*PLA2-IIA*) and L-type calcium channel (*Cacna1c*) genes were upregulated in cardiomyocytes and VSMC after stimulation with both purified antibodies from patients and from rabbit [5]. We showed that patient and rabbit α_1A_-AR-antibodies result in protein kinase C alpha activation and transient extracellular-related kinase (ERK1/2) phosphorylation. The antibodies also exerted acute effects on intracellular Ca^2+^ in cardiomyocytes and contracted mesentery artery segments [5]. In a proof-of-concept study involving the β_1_-AR, Jahns et al immunized rats and showed that agonistic autoantibodies caused idiopathic dilated cardiomyopathy in the same rats. Furthermore, passive transfer also caused disease [6].

Three different receptor subtypes mediate α_1_-adrenergic signaling, namely α_1A_-, α_1B_-, and α_1D_-AR. All subtypes were expressed in cardiac tissue but differ in the amino acid sequence of the second extracellular loop. Zhou et al immunized rats with a second extracellular loop peptide from the α_1D_-AR subtype epitope [7]. The rats developed agonistic antibodies. Tail-cuff systolic blood pressure was not changed. The investigators described cardiac hypertrophy, increase in the collagen deposition, c-*jun*, and matrix metalloproteinase 2 (MMP2) expressions in the heart. We immunized our animals against the second extracellular loop of α_1A_-AR. This report is the first showing the *in vivo* relevance of α_1A_-AR-AB (as opposed to α_1D_-AR-AB) to our knowledge. We investigated the effects on blood pressure by radiotelemetry and on cardiac function by invasive hemodynamic measurements with a conductance catheter and echocardiography. Cardiac molecular pathways influenced by α_1A_-AR-AB signaling were investigated by gene expression array analyses. Furthermore, we tested the hypothesis whether immunized rats react more sensitive to angiotensin (Ang) II.

## Materials and Methods

### Immunization

Experiments were performed in 36 male Lewis rats aged 8 weeks. We prepared a synthetic GWRQPAPEDETICQINEEPGYVLFSAL-AmidxTFA/salt (Biosyntan GmbH, Berlin, Germany) peptide corresponding to the second extracellular loop of human α_1A_-AR. Eighteen rats were immunized by subcutaneous injection (200 µg, treated with 350 µg methylated albumin) dissolved in 1 mL saline at 0, 2, and 4 weeks. The animals were boosted monthly over 12 months. Eighteen control rats received saline. For Ang II infusion, osmotic pumps (Alzet, Cupertino, CA, USA) were implanted under isoflurane anesthesia in the animals (n = 6 per group) 12 months after first immunization. The animals received 200 ng Ang II/kg/min for 14 days (Calbiochem, La Jolla, CA, USA). Local authorities (LAGeSO, Berlin, Germany) approved the animal protocol that complied with criteria outlined by the American Physiological Society.

### α_1_-AR-AB Detection

Rat α_1A_-AR-AB were detected by peptide ELISA (CellTrend, Luckenwalde, Germany). Rat sera (100 µL), 3 or 12 months after first immunization, were added (dilution 1∶1000). As second antibody, we used rabbit anti rat IgG fc horseradish peroxidase (HRP) conjugated (1∶35000 diluted, 100 µL/well, Bethyl, Montgomery, TX, USA). The reaction was detected by tetramethylbenzidine (TMB) as substrate for the enzyme HRP.

Neonatal rat cardiomyocyte contraction assay and the detection of extracellular regulated kinase 1/2 (ERK1/2) phosphorylation in CHO cells stably transfected with human α_1A_-AR (CHO/α_1A_-AR) were carried out as earlier described [5]. For the ERK1/2 phosphorylation experiments, 50 µg of IgG purified from sera of rats 3 months after immunization and controls were added to the CHO/α_1A_-AR cells for 10 min. We checked specificity by inhibiting with 1 µM of α_1_-AR antagonists prazosin or urapidil. The development of AT_1_-AR-AB, β1-AR-AB, or β_2_ AR-AB during immunization or Ang II treatment was excluded by cardiomyocyte contraction assay in presence of the antagonists.

### Echocardiography, Blood Pressure and Hemodynamic Measurements

Rats were anesthetized with 2% isoflurane and kept warm on a heated platform. Temperature and ECG were continuously monitored. Cardiac function and morphology were assessed by echocardiography with a VisualSonics Vevo 770 High-Resolution Imaging System with the use of a high-resolution (37.5 MHz) transducer. The telemetry system (Dataquest ART 4.0™, Data Sciences International, St. Paul, MN, USA) and the implantation procedure is described in detail by Brockway et al. [8]. The radiotelemetry pressure transducers (TA11PA-C20) were implanted in the abdominal cavity of the rat under isoflurane anesthesia, with the transducer connected capillary tubing anchored in the lumen of the abdominal aorta. Before the implantation the zero offset was measured and the unit was soaked in 0.9% NaCl. Animals were allowed to recover for 10 days. The data from the TA11PA-C20 device were transmitted via radiofrequency signals to a receiver below the home cage and thereafter collected (sampling rate 500 Hz). The system monitors mean, systolic and diastolic blood pressure, heart and respiration rate and locomotor activity at 5-min intervals, while the rats move freely. We report mean arterial blood pressure (MAP). For hemodynamic measurements, rats were intubated and ventilated under isoflurane anesthesia. A 2-French conductance catheter (SPR 838 Aria, Millar Instruments, Houston, TX, USA) was inserted into the left ventricle through the right carotid artery. The stroke volume (SV), end-diastolic volume (Vol max), end-systolic volume (Vol min), and LV-pressures were measured directly in acute experiments.

### Gene Expression and Immunohistochemistry

Total RNA was extracted from the cardiac apex (one-third from the whole heart including parts of left, right ventricle and septum) of three immunized and three control rats using the RNeasy Purification Kit (Qiagen GmbH, Hilden, Germany). RNA was treated by deoxyribonuclease I (Qiagen). Two µg RNA of cells were transcribed in cRNA with One-Cycle Target labeling and Control Reagents (Affymetrix, Santa Clara, CA, USA). Non-pooled microarray experiments were performed using Rat Genome 230 2.0 Arrays (31,099 probe sets, Affymetrix). Gene expression and RT-PCR experiments were carried as previously described.[5] Primer sequences for TaqMan analyses are listed in Supplementary table S1. The MIAME-compliant microarray data are available http://www.ebi.ac.uk/arrayexpress/experiments/E-TABM-725.

Cardiac tissues were fixed in paraformaldehyde and embedded in paraffin. The 5 µm thick sections were deparaffinized, rehydrated, and stained by the Trichrome-Masson-Goldner or 0.1% Sirius red saturated in picric acid. Histomorphological analysis and cardiomyocyte diameter was determined on elastica van Gieson and hematoxylin-and-eosin–stained, 4 µm-thick sections of tissue placed in 5% formalin. Cardiomyocyte diameter was determined perpendicular to the outer contour of the cell membrane at the nucleus level in 15 representative myocytes of the section as desribed by van Heerebeek et al [9]. Heart sections were photographed at a magnification of 10x (interstitial) or 20x (perivascular) with a Sony AVT-Horn camera using Zeiss-Axioplan 2 microscope. 25 microscopic view fields (left ventricle and septum) were evaluated. Perivascular fibrosis was analysed from cross sections of coronary arteries (12 arteries per section). Analysis and quantification of interstitial and perivascular fibrosis content were performed with “Image J” software public domaine (Wayne Rasband, NIH, USA). Data are presented as fractional area of fibrosis content in % of myocardial tissue. The investigators performing the analysis were not aware of the experimental groups.

### Statistics

For analysis of echocardiography, telemetric, and hemodynamic measurements Mann-Whitney-test was used at P<0.05. Data are expressed as mean ± SD. Continuous variables (blood pressure and heart rate) were analyzed by repeated measures analysis of variance with appropriate corrections.

## Results

### Immunization and α_1A_-AR-AB Detection

**Figure 1:**
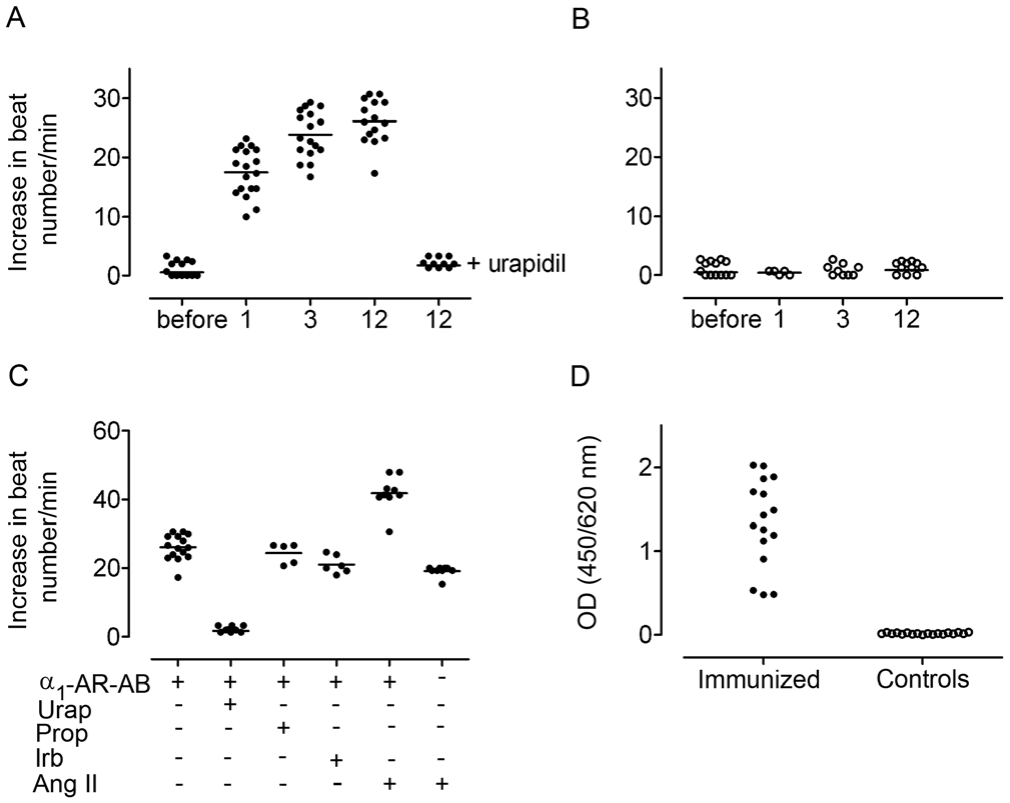
Rat α_1A_-AR-AB detection. (**A** and **B**) Ordinate shows neonatal rat cardiomyocyte spontaneous beating rate; abscissa shows the immunization time point. IgGs from immunized rats increased the beating rate (**A**), compared to controls (**B**) Specificity was checked by inhibition with 1 µM urapidil (Urap). (**C**) We excluded autoantibodies against other receptors using the b1-AR antagonist propranolol (Prop) and the AT1 receptor antagonist irbersartan (Irb). The combination of α_1A_-AR-AB and Ang II increased the cardiomyocyte contraction. (**D**) A high titer of α_1A_-AR-AB was detected by ELISA in immunized rats, compared to controls.

**Figure 2:**
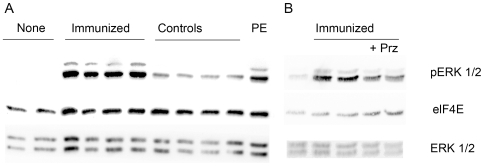
ERK1/2 activation in CHO/α_1A_-AR cells. (**A**) Activation after incubation with IgG from immunized and control rats or with phenylephrine (10 µM) for 10 min is shown. Lane 1 and 2 represent untreated cells. (**B**) The specificity of ERK1/2 activation by α_1A_-AR-AB was checked by prazosin inhibition (Prz, 1 µM). Lane 1 represents untreated cells. Eukaryotic initiation factor 4E (elF4E) and ERK1/2 antibody were used as loading control.

The cardiomyocyte contraction assay documented an increase of α_1A_-AR-AB activity one month after first peptide injection persisting over the immunization process of 12 months. Urapidil inhibited the activity (Figure 1A). IgG fractions eluted from sera of control rats were negative in the cardiomyocyte contraction assay (Figure 1B). The development of autoantibodies against other receptors during immunization or Ang II treatment was excluded by cardiomyocyte contraction assay in presence of the specific antagonists. The combination of α_1A_-AR-AB and Ang II increased the cardiomyocyte contraction, compared to the effect of α_1A_-AR-AB or Ang II alone (Figure 1C). A high titer of α_1A_-AR-AB was detected by ELISA in the immunized rats. The controls did not show a signal (Figure 1D). The incubation of CHO/α_1A_-AR cells with IgG prepared from immunized rats resulted in a stronger ERK1/2 phosphorylation than IgG fractions eluted from sera of control rats (Figure 2A). The specificity was proven by inhibition with the α_1_-AR receptor antagonist prazosin (62% inhibition, P = 0.023, Figure 2B).

### Cardiac Function in Immunized Rats

**Figure 3:**
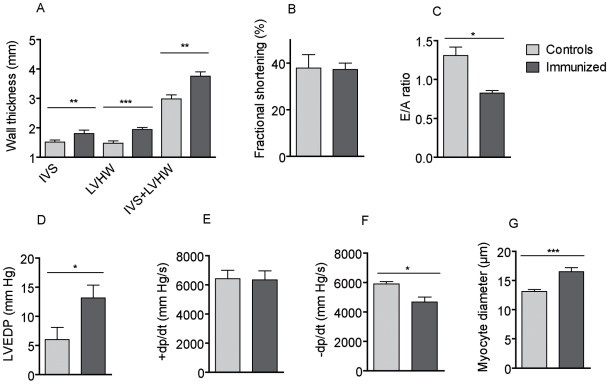
Echocardiography and invasive hemodynamic by conductance catheter at 12 months after first immunization. (**A**) Increased interventricular septum (IVS) and left ventricular heart wall (LVHW) thickness indicates hypertrophy. (**B**) Unchanged fractional shortening was a sign for preserved systolic function. (**C**) A decreased E/A ratio in immunized rats indicated a diastolic dysfunction. (**D**) Immunized rats had increased left ventricular end-diastolic pressure (LVEDP), (**E**) Unchanged dp/dt_max_ and (**F**) diminished dp/dt_min_, indicating impaired relaxation/diastolic function. (**G**) Mean diameter of cardiomyocytes showed a significant hypertrophy, 12 months after immunization with α_1A_-AR peptide compared to controls. * indicates P<0.05, ** indicate P<0.01, *** indicate P<0.001.

**Figure 4:**
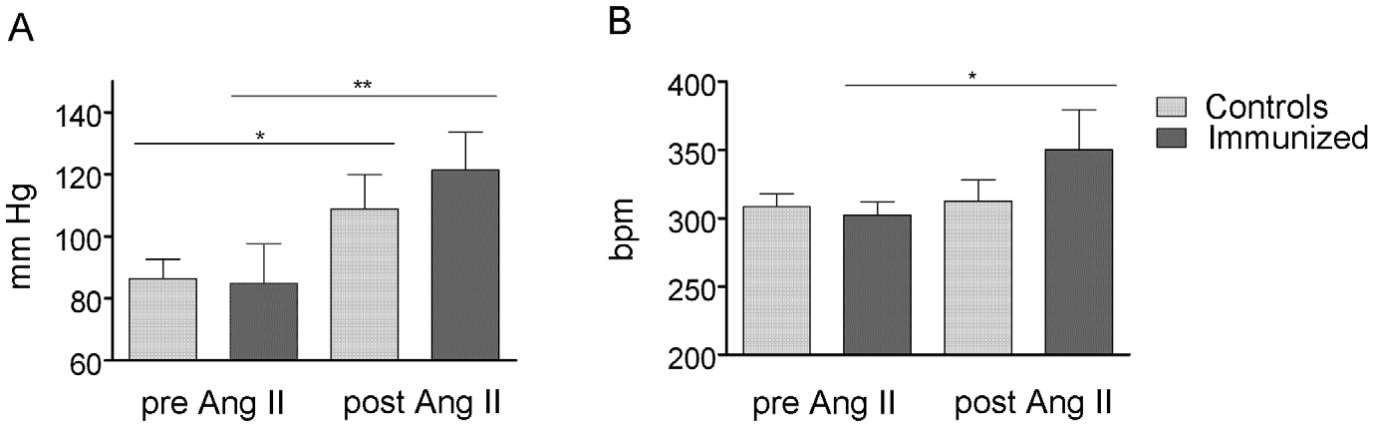
Mean arterial blood pressure (MAP) and heart rate 12 months after first immunization, pre and post angiotensin (Ang) II treatment. α_1A_-AR-AB in combination with the stressor Ang II cause a greater increase in blood pressure (**A**) and heart rate (**B**) in the immunized rats compared to the Ang II-treated controls. * indicates P<0.05, ** indicate P<0.01.

The diastolic interventricular septum and diastolic left ventricular heart wall thickness were significantly increased in 12 months immunized rats compared to the controls (Figure 3 A). The ratio of heart/body weight (HW/BW×1000) was significantly increased in the immunized rats compared to the controls (2.21±0.23 vs. 1.97±0.04, P = 0.0471, Supplementary table S2). Fractional shortening was similar in both groups, so that systolic function was preserved (Figure 3 B). A decreased ratio of peak flow velocity of the early rapid diastolic filling wave to peak flow velocity of the late diastolic filling wave (E/A ratio) in immunized rats indicates diastolic dysfunction (Figure 3 C). The echocardiographic findings in vivo are summarized in Supplementary table S3. Hemodynamic measurements showed higher left ventricular end-diastolic pressures (LVEDP) in immunized rat hearts (Figure 3 D). No changes in dp/dt_max_ confirmed the preserved contractility/systolic function (Figure 3 E) and the decreased dp/dt_min_ indicates an impaired relaxation/diastolic function (Figure 3 F). The analysis of the mean cardiomyocyte diameters showed a significant hypertrophy 12 months after immunization with α_1A_-AR peptide, compared to controls (Figure 3 G). The blood pressure (MAP) and heart rate measured by telemetry were not different 12 months after first immunization (Figure 4).

### Gene Expression Analysis and Histology

Genes coding for proteins of the sarcomere and extracellular matrix were differentially expressed in the heart (Table 1). Thus, the myosin heavy chain alpha (*α-MHC*) and the myosin heavy chain beta (*β-MHC*) were upregulated. Further, the collagen type I maintaining tissue structure and collagen type IV, a component of basal lamina that allows interaction with integrins and favors cell adhesion, were increased expressed. The collagen IV binding glycoprotein laminin was also upregulated. Other genes coding for proteins involved in the Ca^2+^ signaling were represented. Among them were the cardiac ryanodine receptor 2 (*Ryr2*), the cardiac ATPase 2 Ca^2+^-transporting, slow-twitch (*Atp2a2*), and the L-type calcium channel (*Cacna1c*). Important components of the energy metabolism like muscle glycogen phosphorylase and the peroxisome proliferators-activated receptor-gamma, co-activator 1, alpha (*Ppargc1a*) a master regulator of metabolic function responsible for fatty acid uptake and oxidation and oxidative phosphorylation[10] were upregulated (Table 1).

**Table 1:** Selected differentially expressed genes in hearts of immunized rats.

Gene	Ref.-Sequence	Fold change
Sarcomere		
Myosin heavy chain, alpha	NM_017239	2.7
Myosin heavy chain, beta	NM_017240	2.3
Collagens and extracellular matrix proteins		
Collagen, type I, alpha 1	NM_053304	1.5
Collagen, type I, alpha 2	NM_053356	1.6
Procollagen, type IV, alpha 1	XM_001067473	1.7
Procollagen, type IV, alpha 2	XM_001076134	1.5
Laminin, gamma 1	XM_001071300	1.9
Ca^2+^-regulation		
Ryanodine receptor 2, cardiac	XR_006681	1.7
ATPase, Ca^2+^-transporting, cardiac muscle, slow twitch 2	NM_001110139	1.5
Ca^2+^-channel, voltage-dependent, L type, alpha 1C	NM_012517	1.5
Energy metabolism		
Peroxisome proliferators-activated receptor-gamma, coactivator 1, alpha	NM_031347	1.8
Muscle glycogen phosphorylase	NM_012638	1.7
Phosphofructokinase, muscle	NM_031715	1.5

**Figure 5:**
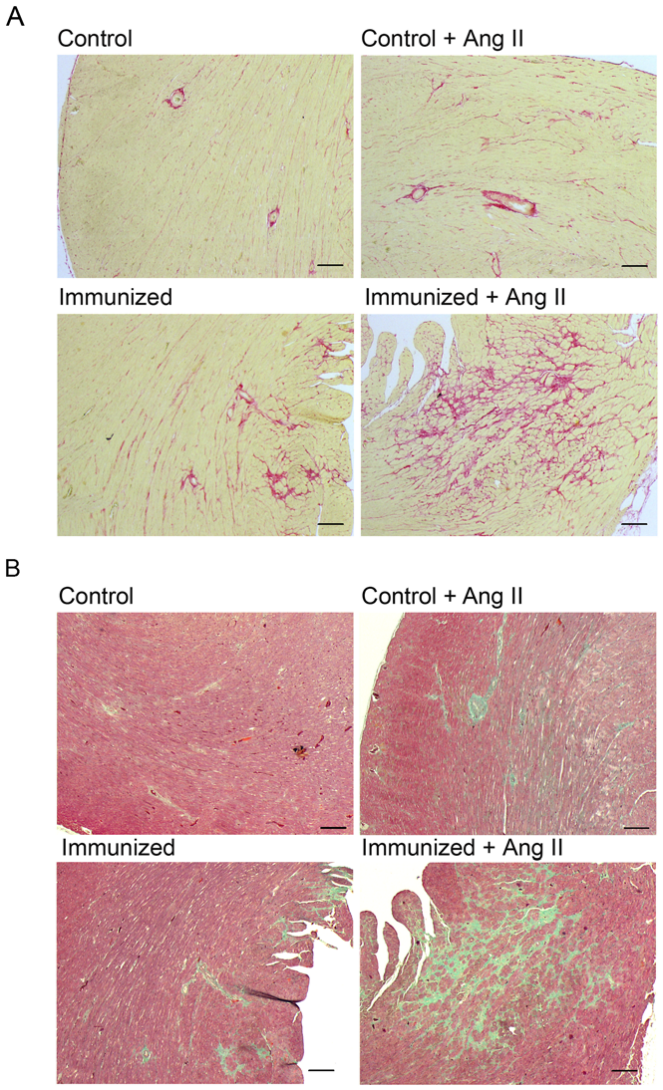
Cardiac remodeling and fibrosis in immunized rats, pre and post Ang II treatment. (**A**) Sirius red and (**B**) Trichrome-Masson-Goldner staining. Both stains showed marked fibrosis in the hearts of immunized rats. α_1A_-AR-AB, in combination with the stressor Ang II cause a significantly higher increase of fibrosis in immunized rat hearts compared to the Ang II treated controls (Bar = 0.2 mm).

**Figure 6:**
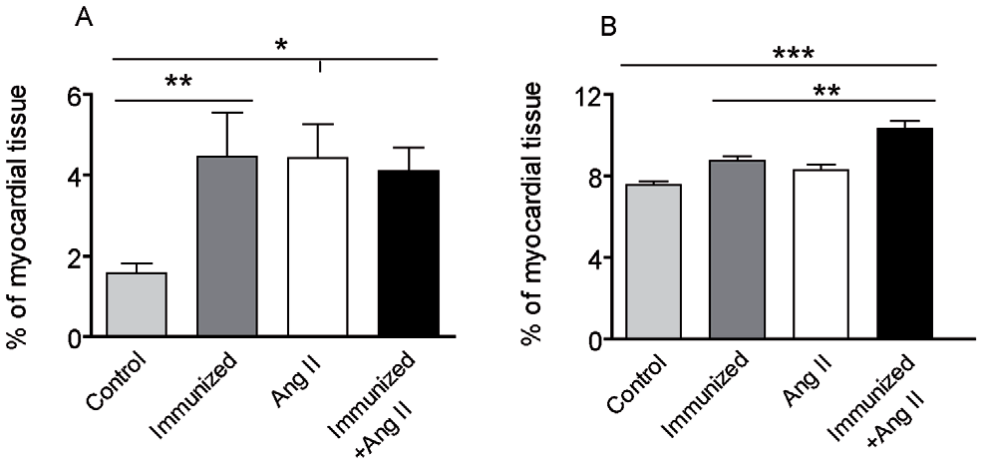
Myocardial perivascular and interstitial collagen. (**A**) Perivascular fibrosis was significantly increased in all three treated groups compared to control rats. (**B**) Interstitial fibrosis was significantly increased in immunized rats treated with Ang II when compared to controls, rats treated with Ang II or immunized rats.

The gene expression results were validated by TaqMan analysis for *α-MHC* (Fold change (FC) = 3.5), *β-MHC* (FC = 2.8), collagen, type I, alpha 1 (FC = 1.7), and *Cacna1c* (FC = 2.0). The cardiac expression of α_1A_-, α_1B_- or α_1D_-AR subtype was not differential in immunized and control rats one year after immunization. Sirius red and Trichrome-Masson-Goldner staining were used for fibrosis detection. More fibrosis occurred in immunized rats compared to the controls (Figure 5 A, B). Perivascular fibrosis was significantly increased by α_1A_-AR-AB, whereas interstitial fibrosis was significantly induced in combination with Ang II infusion (Figure 6).

### Ang II Effects in Immunized and Control Rats

We tested whether or not immunized rats react more strongly to Ang II. Ang II further increased the chronotropic response of α_1A_-AR-AB, whereas phenylephrine did not result in an incremental increase in the bioassay (Supplementary figure S1). The combination of α_1A_-AR-AB and Ang II caused a stronger increase in MAP in immunized rats, compared to the Ang II treated controls (Figure 4, P = 0.0339). The ratio of heart/body weight (HW/BW×1000) was significantly increased in the immunized rats compared to the controls (2.31±0.13 vs. 2.08±0.12, P = 0.0109, Supplementary table S2). We used Trichrome-Masson-Goldner staining to quantify our results and found that immunized rats developed more severe fibrosis than control rats (Figure 5 A, B). The extent of fibrosis as a percent of tissue area was significant higher in the immunized rats (14.4±4.54% vs. 8.90±2.31% in controls, P<0.00001). Ang II also induced a significant myocyte hypertrophy, which was not potentiated by α_1A_-AR-AB (data not shown).

## Discussion

The important findings in our study were that active immunization results in production of activating antibodies to the α_1A_-AR, namely α_1A_-AR-AB. The α_1A_-AR-AB in our model did not increase telemetrically measured blood pressure. However, they clearly caused cardiac target organ damage and resulted in a model of diastolic dysfunction. Finally, stimulation of the system with an additional stressor (Ang II) resulted in worsened target-organ damage in the α_1A_-AR-AB-producing animals compared to controls. The immediate question is, “how do the results reported here differ from those of Zhou et al [7]?” We relied on a different immunization sequence; our sequence corresponded to α_1A_-AR, while Zhou et al used a sequence corresponding to α_1D_-AR. These receptors are different. We investigated Ang II provocations, while they did not. We focused on target-organ damage. Finally, we monitored endorgan damage with echocardiography, invasive hemodynamics, histological analysis, radiotelemetrically determined blood pressure measurements, and by gene expression array.

The immunized rats developed specific α_1A_-AR-AB that we detected by both cardiomyocyte contraction assay and ELISA. The blocking experiments underscored the specificity of these antibodies. The antibodies were capable of initiating α_1A_-AR signaling as documented by our phospho-ERK1/2 experiments. We documented clear-cut differences in cardiac function by two independent techniques, one relying on direct cardiac catheterization that revealed a diastolic pathology. Cardiac structure was substantially different between the groups, as shown by echocardiography and by light microscopy. Our notion that a much more sensitive blood pressure measurement than the tail-cuff technique would show a difference in blood pressure between groups, proved not to be the case. Nonetheless, the Ang II experiments showed clearly that when a driving force for hypertension is applied, the presence of α_1A_-AR-AB clearly aggravates target organ damage and blood pressure. Finally, we explored new mechanistic pathways.

Our gene expression analyses showed different compensatory mechanisms in structure and metabolism to maintain the cardiac function. In contrast to the failing rat heart with a shift in myosin isoform from α to β-MHC [11], we found instead an increased expression of both MHC isoforms. Up-regulation of β-MHC transcription can serve as an early and sensitive marker of cardiac hypertrophy [11], [12] and may conserve energy [13], [14]. Forced expression of α-MHC may be beneficial in terms of increasing the myocardial contractility and may result in cardioprotection [15], [16].

Myocardial remodeling implies an alteration in the extracellular matrix composition and distribution. Accordingly, we found in our array analysis the upregulation of collagen type I and IV in immunized rat hearts. Collagen I and III maintain the tissue structure, transmit forces throughout the myocardium and contribute to the elastic properties of the myocardium [17]. The increased accumulation of collagen I and III has been associated mostly with fibrosis [18], [19]. Type IV and VI collagens are components of the basal lamina and favors cell adhesion. The increased expression could be involved in the alteration of extracellular matrix cell interaction [20]. In human dilated cardiomyopathy, collagen is degraded by metalloproteinases and is replaced by fibrous intercellular deposits [21]. Zhou et al found increased MMP2 expression and activity in their α_1D_-AR-AB immunization study [7]. We did not observe any increased metalloproteinase or tissue inhibitors of metalloproteinases. Other up-regulated genes encode molecular regulators of energy metabolism. The peroxisome proliferator-activated receptor gamma (PPAR-γ) coactivator 1-alpha (*Ppargc1a*) activates multiple genes that are responsible for fatty acid uptake and oxidation and for oxidative phosphorylation [10]. The development of heart failure is accelerated by *Ppargc1a* deficiency [22], suggesting that this coactivator may have a cardioprotective function.

Another important observation is the fact that genes coding for important Ca^2+^ regulating proteins, such as ATPase, Ca^2+^ transporting, slow-twitch (*Atp2a2*) coding for the sarcoplasmic/endoplasmic reticulum calcium ATPase (SERCA2a), the cardiac ryanodine receptor 2, and the L-type calcium channel, alpha 1 C subunit (*Cacnac1c*) were all up-regulated. The overexpression of SERCA2a in diseased hearts has been shown to result in the recovery of contractility [23], [24], [25] and in improved survival, corresponding with an improvement in energy consumption [26]. Furthermore, SERCA overexpression decreases or prevents cardiac hypertrophy [27], [28], [29]. In our earlier study we found that acute administration of purified α_1_-AR-autoantibodies from patients or rabbit α_1A_-AR-AB to neonatal cardiomyocytes affected intracellular Ca^2+^ at two different levels, namely the acute, short-term elevation of intracellular Ca^2+^, and the increased transcript expression of *Cacna1c* [5]. In this study, we also found a long-term upregulation of *Cacna1c*. A link between increased L-type calcium channel, alpha 1 C subunit levels and hypertrophy has also been demonstrated for the human heart [30].

Rysa et al. performed DNA microarray analysis in 12 month-old SHR with manifest hypertrophy, compared to 16–20 month-old SHR with diastolic dysfunction and transition to heart failure [31]. Most of the enhanced genes upregulated in the development of diastolic heart failure encoded for ECM proteins. ECM proteins were also upregulated in our study. However, whereas we found dysregulated transcripts for calcium homeostasis, myofilament contractile proteins, and cardiomyocyte cytoskeleton proteins, these pathways seemed not to play a significant role in the development of diastolic heart failure caused by pressure-overload hypertrophy. The two models and the experimental settings were considerably different. Our α_1A_-AR-immunized rats were only 12 months old, had no hypertension, and no signs of heart failure, whereas the SHR rats were older and had signs of pressure-induced diastolic heart failure. Interestingly, Wallukat et al have shown that SHR develop autoantibodies against the β1 adrenergic receptor that permanently stimulate the receptor, while α_1_-AR-AB have not been found [32], [33].

The α_1_-adrenergic receptors are important to both developmental cardiomyocyte growth and pathological hypertrophy. The α_1A_-AR-AB production induced hypertrophy by causing fibrosis and cardiomyocyte hypertrophy. Patel et al [34] showed that a 28-day infusion of a subpressor norepinephrine dose induced hypertrophy only by stimulating myocyte growth. No fibrosis or signs of diastolic dysfunction was present in their 28-day study. However, we investigated our model for 1 year. Du et al [35] reported that animals transgenic for the α_1A_-AR showed a greater increase in myocardial fibrosis post-myocardial infarction, compared to the non-transgenic control animals. Transgenic lines with an even higher expression level developed progressive cardiac fibrosis.

In spite of subtype-selective agonists and antagonists and gene knockout and transgenic overexpression approaches, the question of which α_1_-AR subtype is involved predominantly in vasoconstrictive responses to sympathomimetic agonists has not been answered. The studies with knockout mice indicate that all subtypes play a role in the blood pressure response to α_1_-agonists and that the dominant contractile α_1_-AR is different in different vascular beds. Although we did not observe an increase in blood pressure by α_1A_-AR-AB, our results do not justify the conclusion that the α_1A_-AR is not involved in the blood pressure control. Our results are in line with results reported by Tanoue et al [36]. These investigators found a major role for α_1A_-AR in maintaining basal blood pressure, whereas other subtypes such as α_1B_-AR and α_1D_-AR were more important in the pressor response to catecholamines.

Our immunized α_1_-AR-AB-producing rats developed an increased LVEDP and diminished dp/dt_min_ in the face of preserved ejection fraction and fractional shortening. This state-of-affairs is termed “diastolic dysfunction” and is a precursor for diastolic heart failure. Half the patients with heart failure fall into this category, notably older women. The prognosis of the condition is no better, if not worse, than systolic heart failure. All medication trials to date have been disappointing. The α_1_-AR blockers have not been studied in detail in the context of diastolic dysfunction. Two smaller studies reported positive effects of α_1_-AR blockers on echocardiographic parameters of diastolic dysfunction. To our knowledge, this experimental model is the first to show that α_1A_-AR receptor stimulation can cause diastolic dysfunction independent of any change in blood pressure. De Blois et al showed that chronic α_1_-AR stimulation increases smooth muscle cell DNA replication is in arterial wall, leading to remodeling after vascular injury [37]. We speculate that alterations in peripheral resistance (pressure-overload) could have been responsible for the diastolic dysfunction we observed. The fact that we were unable to detect any blood pressure increases even with radiotelemetry, suggests the possibility that altered blood pressure buffering played a role. We demonstrated heart muscle cell (cardiomyocyte) hypertrophy, in addition to an increased cardiac fibrosis in the α_1A_-AR-AB model. We believe that these changes, independent of any blood pressure changes we were able to detect, resulted in the diastolic dysfunction that we observed.

Our Ang II experiments showed that Ang II markedly aggravated the already-present cardiac fibrosis. We used Ang II as a stimulus to further induce vascular dysfunction. The sympathetic nervous system and renin-angiotensin-aldosterone system act synergistically to elevate or maintain blood pressure. Ang II signaling plays a critical role in modulating many of the stimuli and signals that govern arterial aging, arterial structural, and vascular functional and adaptational responses. Ang II also potentiated the chronotropic response to α_1A_-AR-AB, whereas phenylephrine infusion, as reported by Patel et al, did not [22].

Limitations in our study are the fact that we did not include a long-term treated control group, namely immunized rats producing α_1A_-AR-AB treated with chronic α_1_-AR blocker therapy. An additional desirable control group could consist of chronic phenylephrine infusion (for 1 year). Acute infusion experiments could elucidate the issue of baroreceptor reflex blood pressure buffering capacity or resetting that remains unanswered from our study. However, our acute experiments showed that the effects of α_1A_-AR-AB could be blocked pharmacologically. Chronic experiments could have allowed us to speculate with greater confidence on a possible role of α_1_-AR blockade to alleviate diastolic heart dysfunction and remodeling.

### Perspectives

We elucidated agonistic autoimmunity-induced target-organ damage and showed that α_1A_-AR-AB after immunization caused diastolic dysfunction. Our animal model suggests that α_1A_-AR-AB could play a role in target-organ damage; however α_1A_-AR-AB are probably not initiators of hypertension. These findings have implications for the notion of viewing agonistic autoantibodies as primary treatment targets in human diseases [38]. Our findings also have implications concerning α_1_-AR blocker therapy. These agents may warrant a closer look, particularly in terms of diastolic dysfunction.

## Supporting Information

Figure S1 Cardiomyocyte contraction assay. The incubation of cardiomyocytes with Î±_1A_-AR-antibodies (AB) or phenylephrine (PE) caused an increase in cardiomyocyte contraction. This effect was not further potentiated by the combination of AB and PE. The combination of AB and Ang II resulted in a further raise of contraction.
(0.13 MB DOC)

Table S1 Primer and probe sequences used for TaqMan RT-PCR.
(0.05 MB DOC)

Table S2 Physiological parameters of immunized and control rats.
(0.03 MB DOC)

Table S3 Echocardiography of immunized and control rats 12 months after first immunization and after Ang II treatment.
(0.04 MB DOC)
