# Supplementary material for: α1A-Adrenergic Receptor-Directed Autoimmunity Induces Left Ventricular Damage and Diastolic Dysfunction in Rats
Source: PLoS One. 2010 Feb 24;5(2):e9409. doi: 10.1371/journal.pone.0009409 (PMC2827566; doi:10.1371/journal.pone.0009409)
Supplement: Figure S1 — Cardiomyocyte contraction assay. The incubation of cardiomyocytes with Î±1A-AR-antibodies (AB) or phenylephrine (PE) caused an increase in cardiomyocyte contraction. This effect was not further potentiated by the combination of AB and PE. The combination of AB and Ang II resulted in a further raise of contraction. (0.13 MB DOC) [file pone.0009409.s001.doc]

Figure S1. Cardiomyocyte contraction assay. The incubation of cardiomyocytes with α_1A_-AR-antibodies (AB) or phenylephrine (PE) caused an increase in cardiomyocyte contraction. This effect was not further potentiated by the combination of AB and PE. The combination of AB and Ang II resulted in a further raise of contraction.
