# Supplementary material for: α1A-Adrenergic Receptor-Directed Autoimmunity Induces Left Ventricular Damage and Diastolic Dysfunction in Rats
Source: PLoS One. 2010 Feb 24;5(2):e9409. doi: 10.1371/journal.pone.0009409 (PMC2827566; doi:10.1371/journal.pone.0009409)
Supplement: Table S1 — Primer and probe sequences used for TaqMan RT-PCR. (0.05 MB DOC) [file pone.0009409.s002.doc]

**Table S1.** Primer and probe sequences used for TaqMan RT-PCR.

| **Gene** |  | **Sequences (5’→3’)** | **Accession No.** |
| --- | --- | --- | --- |
| B2M | for | GCT CGG TGA CCG TGA TCT TT | NM_012512 |
|  | rev | GAG TTT TCT GAA TGG CAA GCA |  |
|  | probe | FAM-TGG TGC TTG TCT CTC TGG CCG TC-TAMRA |  |
|  |  |  |  |
| GAPDH | for | CAA CGG CAC AGT CAA G | NM_017008 |
|  | rev | TCG CTC CTG GAA GAT G |  |
|  | probe | FAM-TGA GAA TGG GAA GCT GGT CAT CA-TAMRA |  |
|  |  |  |  |
| α_1A_-AR | for | CAG TGT CTT CGC AGA AGG CA | NM_017191 |
|  | rev | GGG CGG GTG CAG GGT AT |  |
|  | probe | FAM-TCT TCC AAG CAT GCC CTG GGC-TAMRA |  |
|  |  |  |  |
| α_1B_-AR | for | AGC CCG GCT TCA AGA GC | NM_016991 |
|  | rev | AAA AGG GAC CCT AAA AGT GCC |  |
|  | probe | FAM-ACA TGC CCC TGG CGC CC-TAMRA |  |
|  |  |  |  |
| α_1D_-AR | for | GGC AAG TTT TGG TGG CGT A | NM_024483 |
|  | rev | CCA GAG CGG AAG AGC AAC AG |  |
|  | probe | FAM-TGG ATG CCC GCC GAA CAA A-TAMRA |  |
|  |  |  |  |
| Cacna1c | for | TCA CTG CTG TCG GGA TAA GC | NM_012517 |
|  | rev | GGC CTT CTC CCC TGA AAA G |  |
|  | probe | FAM-AGC TGG GCG GTG TAC GAA GTC G-TAMRA |  |
|  |  |  |  |
| Col1a1 | for | AGA GCG GAG AGT ACT GGA TCG A | NM_053304 |
|  | rev | CTG ACC TGT CTC CAT GTT GCA |  |
|  | probe | FAM-CAA GGC TGC AAC CTG GAT GCC ATC-TAMRA |  |
|  |  |  |  |
| α-MHC | for | CGG GAG AAC CAG TCC ATC CT | NM_017239 |
|  | rev | ACA CGC TTC GTG TTG ACA GTC T |  |
|  | probe | FAM-ATC ACT GGA GAA TCC GGA GCG GG-TAMRA |  |
|  |  |  |  |
| β-MHC | for | GCC AAG ACA GTT CGG AAT GAT AA | NM_017240 |
|  | rev | CCT GTT GCC CCA AAA TGG |  |
|  | probe | FAM-TCC TCC CGA TTT GGG AAA TTC ATT CG-TAMRA |  |

Used annealing temperature was 58°C.
