# Supplementary material for: α1A-Adrenergic Receptor-Directed Autoimmunity Induces Left Ventricular Damage and Diastolic Dysfunction in Rats
Source: PLoS One. 2010 Feb 24;5(2):e9409. doi: 10.1371/journal.pone.0009409 (PMC2827566; doi:10.1371/journal.pone.0009409)
Supplement: Table S2 — Physiological parameters of immunized and control rats. (0.03 MB DOC) [file pone.0009409.s003.doc]

**Table S2.** Physiological parameters of immunized and control rats.

| Treatment | n | Heart weight (g) | Body weight (g) | HW/BW*1000 |
| --- | --- | --- | --- | --- |
| Controls (C) | 6 | 1.17 ± 0.02 | 597.7 ± 17.3 | 1.97 ± 0.04 |
| Controls + Ang II | 6 | 1.17 ± 0.05 | 560.8 ± 14.5 | 2.08 ± 0.12 |
| Immunized (I) | 6 | 1.23 ± 0.04 | 560.3 ± 55.3 | 2.20 ± 0.23 |
| Immunized + Ang II | 6 | 1.28 ± 0.05 | 555.7 ± 17.7 | 2.31 ± 0.13 |
| *P-value* |  |  |  |  |
| I vs. C |  | 0.0248 | 0.1656 | 0.0471 |
| I/Ang II vs. C/Ang II |  | 0.0027 | 0.5660 | 0.0109 |
