# Supplementary material for: α1A-Adrenergic Receptor-Directed Autoimmunity Induces Left Ventricular Damage and Diastolic Dysfunction in Rats
Source: PLoS One. 2010 Feb 24;5(2):e9409. doi: 10.1371/journal.pone.0009409 (PMC2827566; doi:10.1371/journal.pone.0009409)
Supplement: Table S3 — Echocardiography of immunized and control rats 12 months after first immunization and after Ang II treatment. (0.04 MB DOC) [file pone.0009409.s004.doc]

**Table S3.** Echocardiography of immunized and control rats 12 months after first immunization and after Ang II treatment.

|  | Controls | Immunized | P-value | Controls+Ang II | Immunized+Ang II | P-value |
| --- | --- | --- | --- | --- | --- | --- |
| IVSd (mm) | 1.52 ± 0.08 | 1.81 ± 0.11 | 0.00046 | 1.88 ± 0.10 | 1.99 ± 0.19 | 0.3561 |
| LVHWd (mm) | 1.46 ± 0.09 | 1.94 ± 0.06 | 0.0000001 | 1.69 ± 0.10 | 2.03 ± 0.20 | 0.0335 |
| LVDd (mm) | 8.12 ± 0.34 | 7.98 ± 0.60 | 0.9567 | 7.89 ± 0.68 | 7.63 ± 0.72 | 0.6205 |
| LVDs (mm) | 5.17 ± 0.51 | 5.01 ± 0.53 | 0.8936 | 5.15 ± 1.12 | 4.58 ± 0.62 | 0.4192 |
| FS % | 37.93 ± 6.27 | 37.35 ± 2.66 | 0.819 | 35.28 ± 9.0 | 40.09 ± 3.21 | 0.375 |
| EF % | 61.57 ± 6.40 | 65.30 ± 6.67 | 0.3463 | 57.76 ± 5.03 | 57.08 ± 3.62 | 0.5686 |
| MV E (mm/s) | 634.29 ± 243.79 | 556.78 ± 114.58 | 0.510 |  |  |  |
| MV A (mm/s) | 513.19 ± 173.19 | 675.22 ± 116.50 | 0.099 |  |  |  |
| E/A | 1.31 ± 0.30 | 0.82 ± 0.08 | 0.010 |  |  |  |
| DT (ms) | 38.47 ± 9.09 | 41.47 ± 6.40 | 0.5392 |  |  |  |

IVSd: interventricular septum thickness diastolic; LVHWd: left ventricular heart wall thickness diastolic; LVDd: left ventricular diameter diastolic, LVDs: left ventricular diameter systolic; FC: fractional shortening; EF: ejection fraction; MV E: mitral valve peak early filling velocity; MV A: mitral valve velocity at atrial contraction; DT: deceleration time
